# Effects of Use of Primary Care Checklists and of Extent of Clinical Experience on Performance in Interpreting Paediatric ECGs Linked to Risk of Sudden Cardiac Death

**DOI:** 10.3390/healthcare14162628

**Published:** 2026-08-19

**Authors:** Juan Antonio Costa-Orvay, Maria del Carmen Martin-Perez, Emma Gregg Azcarate, Silvia Escriba-Bori, Sergio Verd, Miguel Angel Granados

**Affiliations:** 1 Children’s Heart Unit, Paediatric Department, Can Misses Hospital, 07800 Ibiza, Spain; jacosta@asef.es; 2 Paediatric Unit, Department of Primary Care, Servei Balear de Salut, 07003 Palma de Mallorca, Spain; mariadelcarmen.martin@ibsalut.es (M.d.C.M.-P.); emma.gregg@ibsalut.es (E.G.A.); 3 Children’s Heart Unit, Paediatric Department, Son Espases University Hospital, 07120 Palma de Mallorca, Spain; silvia.escriba@ssib.es; 4 Balearic Islands Health Research Institute (IdISBa), 07120 Palma de Mallorca, Spain; 5 Children’s Heart Unit, Paediatric Department, 12 de Octubre University Hospital, 28041 Madrid, Spain

**Keywords:** checklist, sudden cardiac death, diagnosis, electrocardiography, children, primary care

## Abstract

**Introduction:** Sudden cardiac death in children and adolescents is a devastating yet potentially preventable event. There is, therefore, an urgent need for early recognition of young patients at elevated cardiac risk. Electrocardiographic screening may play a central role in this effort. However, interpreting paediatric ECGs requires advanced diagnostic skills. Checklists have been advocated to mitigate errors in a number of complex fields, both medical and non-medical; however, their effectiveness in interpreting paediatric ECGs remains uncertain. **Objective:** To evaluate whether the use of a structured checklist improves primary care paediatricians’ performance in interpreting paediatric ECGs, and to assess the influence of professional experience on diagnostic accuracy. **Methods:** We conducted a prospective, parallel-group study involving primary care paediatricians in the Balearic Islands (Spain). Participants were randomly assigned to interpret paediatric ECGs, either using routine unstructured interpretation or with checklist support. Outcomes included diagnostic validity ratios, and appropriateness of referral to paediatric cardiologists. Performance was analysed according to checklist use or years of clinical experience. It was also analysed whether diagnostic accuracy varied according to whether the ECG was classified as normal, or abnormal with or without an increased risk of sudden cardiac death. **Results:** Thirty-one paediatricians completed the study, generating 310 ECG interpretations. Checklist use did not significantly improve sensitivity, specificity, or likelihood ratios for detecting ECG abnormalities associated with sudden cardiac death risk, nor did it increase appropriate referral rates. We report a trend towards higher specificity and likelihood ratios among paediatricians with fewer than 20 years of professional experience than among their more senior counterparts. Significantly, this study found true positive rates of normal ECGs, and of abnormal ECGs with risk of sudden cardiac death, to be around 90%, as opposed to true positive rates of around 60% for abnormal ECGs without risk of sudden cardiac death (91% vs. 85% vs. 59%, respectively). **Conclusions:** In this study, checklist support did not enhance diagnostic performance in paediatric ECG interpretation. This finding highlights the need for targeted efforts to improve diagnostic accuracy in this sensitive subset. We also report that mid-career paediatricians appear to achieve the highest ECG diagnostic accuracy, and we show a particularly high rate of correct interpretation of both simple ECGs and high-risk abnormal ECGs.

## 1. Introduction

Sudden cardiac death (SCD) in children is an uncommon event with an estimated incidence which ranges from 0.8 to 6.2 cases per 100,000 children [1,2,3,4]. On the other hand, the loss of a child violates the strongest human bond, deeply rooted in evolutionarily conserved neural circuits, making it different from other types of loss. While only about 10% of individuals develop enduring grief, up to 94% of parents carry long-term grief for a deceased child [5]. The profound impact of paediatric SCD has prompted repeated efforts to detect electrocardiogram (ECG) abnormalities indicative of cardiac disease underlying a risk of childhood SCD. Although screening may be challenging, evidence suggests that this strategy of SCD screening in children yields benefits in terms of long-term health gains [6,7], as demonstrated by the significant reduction in SCD incidence from 3.5 to 1.0 per 100,000 over 20 years following mandatory ECG screening of young athletes in Italy [8].

We previously conducted the ECIAP study to assess the feasibility of screening for SCD-predisposing cardiac disorders in children of 6 and 12 years of age [9]. Although the study proved feasible, the frequency with which paediatricians failed to detect abnormal ECGs associated with SCD risk was concerning. This finding underscores the fact that accurate interpretation of a paediatric ECG places an additional cognitive burden on non-cardiology-trained paediatricians, and serves as a serious reminder that diagnostic error depends heavily on context and is a hidden cause of mortality [10,11]. Although several interventions have been tested, successful interventions to reduce diagnostic error remain elusive [12,13]. One approach shown to be promising for preventing procedural errors across fields from agriculture to aviation [14,15,16] is the checklist. In particular, it has been observed that family medicine residents improve the speed and accuracy of their ECG interpretation when they use a checklist [17].

Checklists ensure that all key variables are assessed by setting out concrete steps, minimising the risk of omission of critical findings. Checklist-based approaches have improved diagnostic reliability in a number of high-complexity medical tasks [18,19,20]. Based on this theoretical framework, we hypothesized that providing primary care paediatricians with a structured checklist of ECG features associated with SCD risk would improve interpretation accuracy compared with usual unstructured reading.

We therefore investigated the primary care paediatricians’ performance in interpreting paediatric ECGs to achieve three main objectives. Our primary objective was to assess whether checklist use increases detection of ECG abnormalities linked to high risk of SCD; the secondary objective was to evaluate whether years of clinical experience influence detection performance; finally, we explored whether specific groups of ECGs, categorized along a continuum from normal tracings to those with high risk, were particularly amenable to interpretation.

## 2. Materials and Methods

### 2.1. Study Design and Participants

We conducted a prospective parallel cohort study in primary care centres across the Balearic Islands (Spain) between December 2023 and March 2024. All primary care paediatricians in the Balearic Islands were invited to participate by an emailed study announcement. The email included an information sheet and an informed consent form for voluntary enrolment. Paediatricians who agreed to participate and returned signed consent were enrolled as study subjects. 

### 2.2. Randomization and Blinding

After enrolment, participants were randomly assigned through a computer-generated sequence in a 1:1 ratio to one of two groups: routine ECG interpretation versus checklist-aided ECG interpretation. Given the nature of the intervention, participants could not be blinded to group assignment. Subsequently, the ECGs were transmitted anonymously, without identifying data of the paediatricians who had interpreted them, to a platform where paediatric cardiologists validated or rejected the primary care paediatricians’ interpretation.

### 2.3. ECG Test Materials

Each paediatrician was asked to interpret the same set of 10 ECGs from children ages 10 to 12, including normal and abnormal findings. The set included 3 normal ECGs, 2 abnormal ECGs with benign abnormalities and no SCD risk (type I second-degree AV block or low atrial rhythm), and 5 abnormal ECGs with SCD risk indicators (long QT syndrome, hypertrophic cardiomyopathy, frequent premature ventricular contractions, Wolff–Parkinson–White syndrome, or third-degree AV block). Digital copies of these 10 ECG tracings were emailed to all participants. Participants in the routine group received a standard answer form to provide their interpretation. Participants in the checklist group received a structured checklist form (Table 1). The process of developing the checklist consisted of predefined steps, following a published integrative approach to ensure its quality [21]. On the basis of the relevant literature and the clinical experience of the authors, a preliminary checklist was developed, pilot-tested, and, finally, submitted to a review round. This was a “content” checklist that provided instruction on systematically interpreting an ECG, and focused on specific ECG features. It consisted of ten key ECG findings related to paediatric SCD risk conditions. All paediatrician participants were asked to classify their findings and to indicate whether a referral to a paediatric cardiologist was indicated in each case.

### 2.4. Data Collection and Outcome Measures

Upon receiving the participants’ interpretations, blinded paediatric cardiologists reviewed the ECG tracings, the paediatricians’ generated diagnoses and the referrals. For each ECG, they specified the correct classification (normal, abnormal without SCD risk, or abnormal with SCD risk), whether referral to a more specialized clinic was warranted, and the specific diagnosis/finding. To assess whether participating paediatricians adequately ruled out or suspected SCD risk, the ECGs were divided into two broad groups: one including normal ECGs and abnormal ECGs without SCD risk; the other including abnormal ECGs associated with SCD risk. The performance of paediatricians who used a checklist was compared with that of those who relied solely on their own expertise. Results were then further stratified by years of clinical experience (<20 vs. >=20 years and <14 vs. >=14 years, the latter corresponding to the sample median). Finally, the overall diagnosis accuracy for normal ECGs, abnormal ECGs without SCD risk (type I second-degree AV block or low atrial rhythm), and ECGs with SCD risk (long QT syndrome, hypertrophic cardiomyopathy, Wolff–Parkinson–White syndrome, or third-degree AV block) was also tested.

### 2.5. Statistical Analysis

A sample of at least 304 interpreted ECG cases (approximately 30 participants reading 10 ECGs each) would provide 80% power (β = 0.20) at a 5% significance level (α = 0.05) to detect a ≥15 percentage point difference in interpretation accuracy between the two groups. This was based on an anticipated baseline accuracy of ~65% in the control group versus ~80% in the checklist group, estimated from prior internal data. With 31 participants and 310 ECG readings, the present study met this target sample size. We agree that repeated observations from the same paediatrician could reduce the effective sample size if substantial within-participant correlation was present. However, each paediatrician interpreted ECGs representing a broad spectrum of unrelated ECG abnormalities, rather than repeated examples of the same condition, making strong within-participant correlation unlikely. Further, to evaluate the potential clustering of observations within paediatricians, we fitted a mixed-effects logistic regression model including paediatrician as a random intercept. The findings would support or discard our use of individual ECG interpretations as the unit of analysis, as accounting for clustering must show how the performed statistical analysis might have materially altered the results. Statistical analyses were performed using SPSS (IBM SPSS Statistics v26) and GraphPad 10.1.1. Binomial calculations for upper and lower bounds of proportions were carried out, as well as chi-square or Fisher’s exact tests, and Student’s *t*-tests were used if applicable. A two-sided *p* < 0.05 was considered statistically significant.

Ethical considerations. This study was approved by the Balearic Islands Clinical Research Ethics Committee (ECIAP-2023). Written informed consent was obtained from all participants. The study was conducted as an investigator-initiated academic project without external funding, in accordance with the Declaration of Helsinki.

## 3. Results

Participant Characteristics. A total of 37 eligible physicians responded to the invitation; of these, 31 (84%) completed the study (the remaining six did not submit interpretations or declined after initial consent). The participants’ median professional experience was 13 years, with 42% having ≤10 years of experience, 29% having 11–20 years, and 29% having >20 years. By random assignment, 14 individuals were allocated to the routine interpretation group and 17 to the checklist group. The two groups did not differ significantly in mean years of experience. Each participant interpreted 10 ECGs, for a total of 310 ECG readings analysed (140 in the routine group, 170 in the checklist group). All participants completed the interpretation of all 10 ECGs assigned. The estimated between-paediatrician variance for appropriate/inappropriate referral was 0.042 (SD = 0.205), corresponding to an intraclass correlation coefficient of 0.013. Thus, only approximately 1.3% of the total variability in ECG appropriate referral was attributable to differences between paediatricians. Given the average cluster size of 10 ECGs per paediatrician, the resulting design effect was 1.11, indicating minimal within-paediatrician clustering.

Main Outcomes: Checklist vs. Routine Interpretation. Overall, the introduction of the checklist did not significantly improve the ability of primary care paediatricians to detect ECG abnormalities associated with paediatric SCD risk. There were no significant differences between groups regarding sensitivity, specificity, or likelihood ratios (Table 2). Likewise, the rate of appropriate referrals to paediatric cardiologists did not vary with checklist use or with paediatricians’ years of experience (Table 3). There was a trend towards lower diagnostic accuracy among senior paediatricians (*p* = 0.108); specificity, sensitivity, and predictive values were around 80% for physicians with <20 years of experience, compared with rates around 70% for those with 20 years of experience or more (Table 4). However, there were no significant differences in diagnosis accuracy among paediatricians with more or less than 14 years of professional practice (this figure corresponds to the sample median) (*p* = 0.654) (Table 5). Finally, we found specificity, sensitivity, and predictive values of around 90% for abnormal ECGs with SCD risk and for normal ECGs, as opposed to values of around 60% for abnormal ECGs without SCD risk. That is, the accuracy of interpretation was high and similar for abnormal ECGs associated with SCD risk and for normal ECGs (*p* = 0.205), whereas the accuracy of interpretation of abnormal ECGs without SCD risk was significantly lower than in both of the abovementioned extreme categories (*p* < 0.0001) (Table 6).

## 4. Discussion

In this prospective study on skills of primary care paediatricians, the use of a checklist for ECG classification did not significantly improve detection of electrocardiographic abnormalities associated with increased risk of SCD, and did not favour more appropriate referral to paediatric cardiologists, when compared with unstructured interpretation. These findings contrast with our initial hypothesis and with evidence from other areas of clinical medicine where checklists have consistently enhanced diagnostic reliability.

Several factors may explain the absence of a measurable benefit of checklist assistance in this setting. Checklists may be effective tools for preventing omissions in complex tasks; however, checklist support alone may be insufficient when the core limitation is insufficient domain-specific expertise. This statement is consistent with reports in the literature indicating that checklist use reduces diagnostic error, particularly among ECG interpretation experts [22,23]. In addition, the checklist used in this study was intentionally brief, which may have limited its capacity to serve as an effective educational scaffold. Finally, checklists can paradoxically induce false positives by restricting focus to the listed items, resulting in other characteristics being missed [24]. The addition of contextual data might have been required in this case to avoid these unintended consequences of checklists [25].

An additional finding was that more recent training may confer an advantage that is not fully offset by accumulated clinical experience. Paediatricians with fewer than 20 years of experience demonstrated a trend towards higher accuracy ratios than their more senior counterparts. This observation aligns with evidence reporting that novice clinicians disproportionately benefit from checklists [23] and that clinicians trained more recently demonstrate greater adherence to updated diagnostic criteria [26], particularly in areas of medicine where knowledge evolves rapidly. Paediatric ECG interpretation is one such area, having improved with clearer definitions of pathological findings over the past two decades. Further, within the gradient of paediatric illness classification, less-experienced clinicians tend to balance detection with uncertainty, whereas experienced clinicians are very good at ruling out severe disease, but at the risk of missing subtle early signs of severe disease [27]. Finally, no significant differences were observed when experience was dichotomized at 14 years, suggesting that the effect of professional seniority on ECG interpretation accuracy may be non-linear and more apparent at longer practice durations.

Our finding of 90% diagnostic accuracy for normal ECGs and for ECGs with SCD risk (long QT syndrome, WPW syndrome, and hypertrophic cardiomyopathy), irrespective of interpretation method or years of experience, warrants ECG screening in this field of public health relevance. One of these findings concurs with previous reports showing that checklists do not negatively impact the diagnosis of normal or simple ECGs [18].

This study has several limitations. The size of the sample of participating paediatricians was modest, although the total number of ECG interpretations did meet predefined power requirements. In addition, the study interpretation of static ECG tracings outside the context of a clinical encounter may not reflect real-world decision making.

In conclusion, we report that more recent training of paediatricians may confer an advantage, and that diagnostic accuracy for both normal ECGs and abnormal ECGs with SCD risk appears to be high. However, given that paediatric SCD remains a devastating yet potentially preventable event, at the same time as AI-assisted ECG technologies are expanding from conventional waveform interpretation to reliable identification of electrophysiological patterns associated with cardiovascular risk [28], future research should evaluate the design of checklists and other interventions that go beyond checklist implementation alone, together with their application to physicians with different levels of expertise and experience.

## Figures and Tables

**Table 1:** Checklist.

ECG Finding	Differential Diagnoses	Notes/Comments	Yes/No
QRS complex			
1. P-QRS correlation preserved	Third-degree (complete) atrioventricular block	—	☐
2. R wave height ≥25 mm in V6 or S-wave depth >25 mm in V1 or S + R in V1 + V5 or V6 (largest value) >35 mm	Hypertrophic cardiomyopathy	—	☐
3. Pathological Q wave depth >3 mm (except aVR and V1) and/or width >0.04 s in at least 2 contiguous leads	Hypertrophic cardiomyopathy Anomalous origin of the left coronary artery (LCA) from the pulmonary artery	—	☐
4. QRS duration > 100 ms and/or delta wave	Wolff–Parkinson–White pattern Arrhythmogenic right ventricular cardiomyopathy (ARVC)	WPW-type pre-excitation: delta wave ± short PR (<0.12 s) ± wide QRS ± ST-T discordant to the delta wave (if most ventricular myocardium is activated via the accessory pathway). ARVC: epsilon waves in right precordial leads.	☐
Repolarisation			
5. ST segment elevation > 2 mm in ≥2 contiguous leads (exclude early repolarisation)	Myocarditis Brugada syndrome Anomalous origin of the LCA from the pulmonary artery	Early repolarisation (normal variant in healthy adolescents): ST elevation < 4 mm with J-point in V4–V6, I, II and aVF. Brugada syndrome ○ Type 1 (“shark-fin”): ST elevation ≥ 2 mm plus T-wave inversion in >1 right precordial lead (V1–V2). ○ Type 2 (“saddle-back”): ST elevation ≥ 2 mm in right precordials with upright or isoelectric T wave.	☐
6. ST segment depression > 0.5 mm in ≥2 contiguous leads	Hypertrophic cardiomyopathy	—	☐
7. Inverted T waves in I, II, aVF, aVL, V4, V5 or V6 in ≥2 contiguous leads	Hypertrophic cardiomyopathy Myocarditis Anomalous origin of the LCA from the pulmonary artery (only in aVL)	Juvenile pattern (normal variant): negative T in V1 that may extend to V2–V3 in younger children; T waves become progressively positive with age. ARVC: negative T waves ± epsilon wave ± wide QRS in V1–V3.	☐
8. QRS–T-axis angle > 90° (especially if T-axis itself is abnormal)	Hypertrophic cardiomyopathy Myocarditis	—	☐
9. Corrected QT (QTc) >0.44 s in males/ >0.46 s in females	Long QT syndrome Myocarditis	—	☐
10. QTc < 0.36 s in both sexes	Short QT syndrome	—	☐

Abbreviations: ARVC—arrhythmogenic right ventricular cardiomyopathy; J-point—junction of QRS and ST segment; LCA—left coronary artery; QTc—heart-rate-corrected QT interval (e.g., Bazett’s formula); WPW—Wolff–Parkinson–White.

**Table 2:** Comparison of the accuracy of ECG interpretation between paediatricians who used a checklist and those who used an unstructured reading approach to get their results.

Statistic	Checklist-Assisted (N = 170 ECGs)		Routine Approach (N = 140 ECGs)		*p*-Value
	Value	95% CI	Value	95% CI	
Sensitivity	72.50%	61.38% to 81.90%	79.17%	67.98% to 87.84%	0.33
Specificity	83.33%	72.70% to 91.08%	79.75%	69.20% to 87.96%	0.55
Positive Likelihood Ratio	4.35	2.55 to 7.42	3.91	2.48 to 6.15	0.72
Negative Likelihood Ratio	0.33	0.23 to 0.48	0.26	0.16 to 0.42	0.49

Abbreviations: CI—confidence interval; ECG—electrocardiogram.

**Table 3:** Comparison of the accuracy of referrals to the Paediatric Cardiology clinic between mid-career and senior paediatricians, and between paediatricians who used a checklist and those who used an unstructured reading approach.

Variable	Paediatricians With Under 20 Years’ Practice	Paediatricians With Over 20 Years’ Practice	*p*-Value	Checklist- Assisted	Routine Approach	*p*-Value
Adequate Referral	173	59	0.680	111	121	0.841
Inadequate Referral	49	19		33	34	

Diagnostic ECG interpretation accuracy of participant paediatricians according to their years of medical practice.

Abbreviations: CI—confidence interval; ECG—electrocardiogram.

**Table 4:** Diagnostic ECG interpretation accuracy of participant paediatricians according to their years of medical practice.

Statistic	≥20 Years’ Experience (N = 80 ECGs)		<20 Years’ Experience (N = 230 ECGs)		*p*-Value
	Value	95% CI	Value	95% CI	
Sensitivity	72.97%	55.88% to 86.21%	80.34%	71.98% to 87.11%	0.341
Specificity	74.36%	57.87% to 86.96%	83.33%	74.94% to 89.81%	0.221
Positive Likelihood Ratio	2.85	1.61 to 5.03	4.82	3.13 to 7.42	0.148
Negative Likelihood Ratio	0.36	0.21 to 0.64	0.24	0.16 to 0.34	0.209
Positive Predictive Value	72.97%	60.44% to 82.67%	83.93%	77.24% to 88.93%	0.139
Negative Predictive Value	74.36%	62.34% to 83.55%	79.65%	72.88% to 85.07%	0.490

Abbreviations: CI—confidence interval; ECG—electrocardiogram.

**Table 5:** Diagnostic ECG interpretation accuracy of participant paediatricians according to their years of medical practice (below or above the median age of this sample).

Statistic	≥14 Years’ Experience (N = 140 ECGs)		<14 Years’ Experience (N = 170 ECGs)		*p*-Value
	Value	95% CI	Value	95% CI	
Sensitivity	78.57%	67.13% to 87.48%	78.82%	68.61% to 86.94%	0.97
Specificity	83.08%	71.73% to 91.24%	84.93%	74.64% to 92.23%	0.75
Positive Likelihood Ratio	4.64	2.67 to 8.07	5.23	3.00 to 9.12	0.79
Negative Likelihood Ratio	0.26	0.16 to 0.41	0.25	0.16 to 0.38	0.94
Positive Predictive Value	83.33%	74.21% to 89.68%	85.90%	77.75% to 91.39%	0.65
Negative Predictive Value	78.26%	69.41% to 85.10%	77.50%	69.33% to 84.00%	0.91

Abbreviations: CI—confidence interval; ECG—electrocardiogram.

**Table 6:** Proportion of true positive results for normal cases, cases without SCD risk and cases with SCD risk.

	Normal ECGs	*p*-Value	ECG Abnormalities Without SCD	*p*-Value	ECG Abnormalities with SCD
Total number/true positive	90/82	<0.0001	62/37	<0.0001	123/105
True positive proportion (95% CI)	0.911 (0.832 to 0.960)		0.596 (0.464 to 0.719)		0.853 (0.778 to 0.910)

Abbreviations: CI—confidence interval; ECG—electrocardiogram; SCD—sudden cardiac death.

## Data Availability

The raw data supporting the conclusions of this article will be made available by the authors on request.
